# Transcriptome Responses of Atlantic Salmon (*Salmo salar* L.) to Viral and Bacterial Pathogens, Inflammation, and Stress

**DOI:** 10.3389/fimmu.2021.705601

**Published:** 2021-09-21

**Authors:** Aleksei Krasnov, Lill-Heidi Johansen, Christian Karlsen, Lene Sveen, Elisabeth Ytteborg, Gerrit Timmerhaus, Carlo C. Lazado, Sergey Afanasyev

**Affiliations:** ^1^Fish Health Department, Nofima AS, Ås, Norway; ^2^Laboratory of Neurophysiology and Behavioral Pathology, I. M. Sechenov Institute of Evolutionary Physiology and Biochemistry, Saint-Petersburg, Russia

**Keywords:** Atlantic salmon, transcriptome, meta-analysis, virus, bacterial pathogen, inflammation, stress

## Abstract

Transcriptomics provides valuable data for functional annotations of genes, the discovery of biomarkers, and quantitative assessment of responses to challenges. Meta-analysis of Nofima’s Atlantic salmon microarray database was performed for the selection of genes that have shown strong and reproducible expression changes. Using data from 127 experiments including 6440 microarrays, four transcription modules (TM) were identified with a total of 902 annotated genes: 161 virus responsive genes – VRG (activated with five viruses and poly I:C), genes that responded to three pathogenic bacteria (523 up and 33 down-regulated genes), inflammation not caused by infections – wounds, melanized foci in skeletal muscle and exposure to PAMP (180 up and 72 down-regulated genes), and stress by exercise, crowding and cortisol implants (33 genes). To assist the selection of gene markers, genes in each TM were ranked according to the scale of expression changes. In terms of functional annotations, association with diseases and stress was unknown or not reflected in public databases for a large part of genes, including several genes with the highest ranks. A set of multifunctional genes was discovered. Cholesterol 25-hydroxylase was present in all TM and 22 genes, including most differentially expressed matrix metalloproteinases 9 and 13 were assigned to three TMs. The meta-analysis has improved understanding of the defense strategies in Atlantic salmon. VRG have demonstrated equal or similar responses to RNA (SAV, IPNV, PRV, and ISAV), and DNA (gill pox) viruses, injection of bacterial DNA (plasmid) and exposure of cells to PAMP (CpG and gardiquimod) and relatively low sensitivity to inflammation and bacteria. Genes of the highest rank show preferential expression in erythrocytes. This group includes multigene families (gig and several trim families) and many paralogs. Of pathogen recognition receptors, only RNA helicases have shown strong expression changes. Most VRG (82%) are effectors with a preponderance of ubiquitin-related genes, GTPases, and genes of nucleotide metabolism. Many VRG have unknown roles. The identification of TMs makes possible quantification of responses and assessment of their interactions. Based on this, we are able to separate pathogen-specific responses from general inflammation and stress.

## Introduction

Since its inception at the beginning of the new millennium, transcriptomics has continued to play an essential role in fish biology and aquaculture research. The assembly and publication of the Atlantic salmon (*Salmo salar* L.) genome (1) have enhanced further development by standardizing gene nomenclature and provided a framework for mapping differentially expressed genes, thereby facilitating comparison among experiments. The number of sequenced bony fish genomes is rapidly increasing, and currently, 77 full genome builds are deposited in Ensembl (https://www.ensembl.org/index.html). However, functional annotations lag far behind the genomes sequencing, assembly, and identification of genes. Migrating the annotations of putative homologues is an efficient and straightforward approach, but it comes with limitations and challenges. The roles of many genes remain unknown even in the best-studied species and the presumed homology and structural similarities do not always imply preservation of functions in vertebrate evolution. Many teleost fish-specific genes remain completely unexplored, despite their active participation in various processes and responses. Transcriptome data is a valuable source of functional annotations, which still hasn’t realized the potential. The main conditions for the association between a gene and a trait or biological process are the reproducibility of responses and the magnitude of differential expression, and meta-analysis of data obtained with RNA-seq and DNA microarrays is the most appropriate strategy for approaching this problem. In addition to the annotation of genes, such a strategy works for the discovery of marker genes. Despite the rapid development of high-throughput methods, qPCR analyses of single genes or small diagnostic sets remain important. Candidate marker genes are often selected based on general knowledge of their roles without detailed information on the expression profiles. Meta-analyses facilitate the discovery of genes with strong and reproducible responses. Identification of co-regulated genes, which we refer to as transcription modules (TMs) enhances the reliability of diagnostics and reduces the risk of misinterpretation of findings. TMs include genes with various functions co-activated under specific conditions, and their composition adds to the understanding of biological processes, and strategies of adaptation and defense.

This report summarizes our research over the last decade since the launch of Nofima’s Atlantic salmon DNA oligonucleotide microarrays (2). Data from a large number of *in vivo* and *in vitro* studies of fish responses to viral and bacterial pathogens, inflammation, wound healing, and stress have reached a critical mass, which makes reliable identifications of TMs possible. The genes were ranked and divided into groups according to the scale of expression changes. Both specialized and multifunctional genes were identified. TMs enable the quantitative assessment of complex transcriptome responses and their separation into biologically meaningful components. Identification and ranking by the stability and magnitude of differential expression aids in selection of candidate marker genes for diagnostics. The first practical outcome of this activity was the development of multi-gene expression assay for assessment of the immune status of Atlantic salmon smolts and growers (3).

## Material and Methods

Nofima’s bioinformatic pipeline STARS (4) is used to process and manage transcriptome data and functional annotation of fish genes. It currently houses 127 experiments with Atlantic salmon that used 6440 microarrays, mainly SIQ-6 (GPL1655 and 30031) containing probes to 15k genes selected by their expression profiles and the functional roles, and 44k genome-wide Salgeno (GPL28079 and 28080) with probes to all identified protein-coding genes. In each experiment, we save one or several lists of differentially expressed genes that fit the standard criteria (>1.75-fold and *p* < 0.05) and contrasts – expression differences, such as differences between the treatment and control groups, time-points, developmental stages etc. Contrasts from exemplary studies with large gene expression changes served as material for the search for co-regulated genes. Microarray data (signal intensity) were also used for the assessment of tissue distribution of transcripts. We used experiments that were published or submitted to NCBI GEO Omnibus (**Table 1**). These trials are described in detail elsewhere, so here we restrict ourselves with brief summaries. The trials were divided into four groups, and four TMs were compiled.

**Table 1:** Summary of studies included in search of transcription modules (TM).

Pathogens, challenges	Cells, tissue*	Time-points	Reference
***Virus responsive genes***			
Salmonid alphavirus	Heart	21 days	(5), GSE173130, GSE183260
Piscine orthoreovirus	Heart	42, 56, 63 and 77 days	(5), GSE183260
Piscine orthoreovirus	Erythrocytes	35 and 42 days	(6), GSE183005
Salmon gill poxvirus^1^	Gill	8 and 12 days	(7 ), GSE151463
Infectious pancreatic necrosis virus	Liver, TO cells	4, 6, 8 and 10 days	GSE172862
Infectious salmon anaemia virus	ASK cells	1 and 5 days	GSE183265
Poly I:C	Adipocytes,	1 and 4 days	GSE171562
Poly I:C	ASK cells	1 and 5 days	GSE183265
***Bacterial diseases***			
*Moritella viscosa*	Head kidney, skin, spleen	42 days	GSE173130
*Moritella viscosa*	Skin, spleen	36 days	(8), GSE171693
*Moritella viscosa*	Skin (epidermis and dermis)	4 and 35 days	GSE171738
*Tenacibaculum finnmarkense*	Skin, dermis, epidermis	3 days	GSE171699
*Piscirickettsia salmonis*	Head kidney	60 days	GSE173095
***Inflammation***			
PAMP (CpG, gardiquimod)	Mononuclear phagocytes	7 days or 6 + 1 day	(9), GSE126993
Wound	Skin	1, 3, 7 14 days	(10, 11), GSE122142
Plasmid DNA	Skeletal muscle	1, 2 weeks	(12, 13), GSE106111
^2^ Melanized foci	Skeletal muscle		(14), GSE182962
***Stress***			
Exhaustive exercise	Heart, spleen	2 hours	GSE173119
Cortisol implant	Skin	18 days	(15), GSE36072
Crowding	Skin	1 day	GSE173229

^1^Field material from outbreak. All other data on virus and bacteria infected fish are obtained in challenge trials.

^2^Field material (slaughter fish).

-Virus responsive genes (VRG). The research included five pathogens (four RNA and one DNA virus): salmonid alphavirus (SAV) causing pancreas disease (PD) (16), piscine orthoreovirus (PRV) causing heart and skeletal muscle inflammation (HSMI) (17), the recently identified salmon gill poxvirus (SGPV) causing gill disease (18), infectious pancreatic necrosis virus (IPNV) causing infectious pancreatic necrosis (IPN) and infectious salmon anaemia virus (ISAV), causing infectious salmon anaemia (ISA). Double-stranded synthetic RNA polyinosinic: polycytidylic acid (poly I:C) is a widely used synthetic analog capable of inducing strong antiviral responses (19).

-Bacteria responsive genes (BACT). We have performed challenges with three bacteria: *Moritella viscosa*, the aetiological agent of winter-ulcer disease (20), *Tenacibaculum finnmarkense* inducing skin lesions and mouth erosions (21, 22), and *Piscirickettsia salmonis* (23), causing piscirickettsiosis, a severe disease with high mortality.

-Inflammation responsive genes (INFL). The main data sets were time-course of wound healing in skin and melanized foci in skeletal muscle. In addition, we included exposures of primary cultures of head kidney leukocytes to PAMP – TLR agonists CpG oligodeoxynucleotides and gardiquimod, and analyses of skeletal muscle injected with bacterial DNA (plasmids).

-Stress responsive genes (STR). A suite of genes was activated in the heart and spleen after acute swimming exercise, in the skin after crowding, and in the skin of salmon with cortisol implants.

The meta analyses were performed in three stages. In trials with bacterial and viral challenges, infected tissues with strong expression changes were selected (tissue that do not harbor pathogens show only weak unspecific responses). The log_2_-expression ratios were first averaged across trials and then between trials with the same pathogen or treatment. In several trials with few contrasts and high variance, medians were calculated instead of means. Genes that showed significant differential expression in several independent trials and higher than 2-fold mean expression changes were included in TMs. To rank the genes, the magnitude of expression changes in each TM was divided into three (VRG, BACT, and INFL) or two (STR) equal intervals. The list of genes with ranks is in **Supplementary File**. Enrichment analysis compared the numbers of genes per functional category (GO) and pathway (KEGG) in TM and on the genome-wide Salgeno platform. Significance was assessed with Yates’ corrected chi-square (*p* < 0.05).

## Results

### An Overview

**Figure 1:**
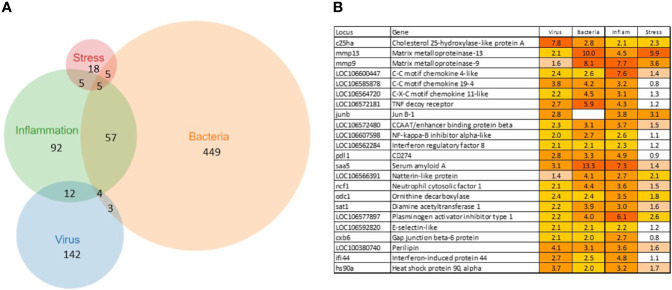
Transcription modules. **(A)** numbers of up-regulated genes and overlap between TMs. The down-regulated genes are specific for each TM, no sharing between TMs. **(B)** multifunctional genes assigned to at least three TMs.

A total of 902 Atlantic salmon genes were annotated (**Supplementary File 1**). All down-regulated and most of the upregulated genes were assigned to only one TM (**Figure 1A**), suggesting relatively high specificity of responses. However, there was some overlap, smallest for VRG and largest for INFL and a suite of multifunctional genes involved in more than one TM was found (**Figure 1B**). One gene, *cholesterol 25-hydroxylase a*, was included in all TMs, though its responses to viruses were much greater than to other challenges. This enzyme, which is induced with infection and inflammation (24), restricts the propagation of viruses, including ARS-CoV-2 (25). Of 22 genes present in three TMs, the greatest expression changes have been shown by two matrix metalloproteinases, *mmp 9* and *13*, suggesting remodeling of extracellular matrix as an essential strategy of salmon responses to pathogens and stressors. The list includes three *chemokines* and *tnf decoy receptor*, which showed a high correlation with histopathology in SAV infected heart (5). Two pleiotropic transcription factors (*jund1* and *ceb/p beta*) and two immune regulators (*irf8* and *nfkb inhibitor*) can orchestrate expression changes of multiple genes. Of acute phase proteins, the structure and functions of *serum amyloid a* are well conserved in all vertebrates (26), while *natterin-like* proteins are specific for fish (27). The multifunctional genes also included free radicals producing *neutrophil cytosolic factor 1*, two enzymes of polyamine and nitric oxide metabolism (*ornithine decarboxylase* and *diamine transferase*), regulators of cell adhesion *plasminogen activator inhibitor* and *e-selectin* and chaperone *hsp90 alpha*. The immune role of *perilipin*, a protein of lipid droplets, to our knowledge, has not been reported in fish. Enrichment analysis was performed in four TMs. Most part of the enriched GO terms and KEGG pathways are associated with the immune and related systems (e.g. angiogenesis, hemopoiesis), and encompass communication, signal transduction, cell differentiation and migration, humoral and cellular effectors (**Table 2**).

**Table 2:** Enrichment of functional categories (GO) and pathways (KEGG).

Functional group/pathway	VRG	BACT	INFL	Total
*Immune responses*				
Defense response to virus	43	16	8	522
TNF-mediated signaling pathway	8	NS^1^	NS	293
Toll-like receptor signaling pathway	7	NS	NS	230
Blood coagulation	6	NS	NS	537
Cytokine-mediated signaling pathway	14	41	20	764
Cytokine activity	NS	12	NS	309
Lipoxygenase pathway	NS	7	NS	38
Jak-STAT signaling pathway	8	11	8	288
B cell receptor signaling pathway	NS	9	6	230
Acute-phase response	NS	11	5	90
Complement and coagulation cascades	NS	14	NS	142
Fc gamma R-mediated phagocytosis	NS	NS	10	269
Fc-epsilon receptor signaling pathway	NS	11	9	420
Inflammatory response	NS	63	17	986
Platelet degranulation	NS	15	10	438
Antigen processing and presentation	NS	9	NS	129
Chemotaxis	NS	18	6	310
Leukocyte cell-cell adhesion	NS	14	7	117
Myeloid cell differentiation	NS	5	NS	113
*Other systems. Signaling & differentiation*				
Cell surface receptor signaling pathway	NS	24	10	746
Insulin receptor signaling pathway	NS	8	NS	289
Integrin-mediated signaling pathway	NS	15	10	400
VEGF receptor signaling pathway	NS	13	11	301
Extracellular matrix organization	NS	21	NS	917
Hematopoietic cell lineage	NS	15	6	132
Angiogenesis	NS	28	14	1203
Myelin sheath	NS	9	NS	227
*Cellular processes*				
Ubiquitin-dependent protein catabolism	16	NS	NS	854
Ubiquitin protein ligase activity	10	NS	NS	650
Lipid particle	7	NS	NS	296
Histone binding	6	NS	NS	399

^1^NS, not significant.

### Virus Responsive Genes

**Figure 2:**
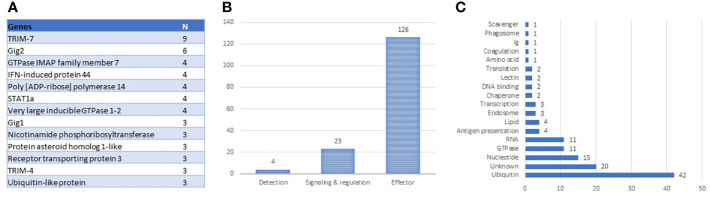
Composition of antiviral TM (VRG). **(A)** genes with more than two paralogs and multi-gene families. **(B)** tiers of antiviral responses with numbers of genes. **(C)** distribution of antiviral effectors by roles.

The antiviral responses of salmonid fish have been described better than any other functional group of the immune system, for a review, see (28–30). The identification of VRG is facilitated by strong and highly reproducible responses to viruses and dsRNA. Transcriptome analyses performed by our (4) and K. Miller’s teams (31, 32) revealed the core set of Atlantic salmon VRG, and the body of knowledge has increased over the past decade. The construction of the genome-wide Salgeno platform expanded the search, while multiple challenge trials with different pathogens provided a more rigorous selection of genes with emphasis on stability of responses. The ranking of 161 VRG by the mean expression changes outlined the core: 19 and 40 genes with correspondingly high and intermediate ranks. This group includes two large multi-gene families: *trim-7* and fish-specific *gig-2* (33) and many paralogs (**Figure 2A**), suggesting duplication and diversification of antiviral genes as the main evolutionary scenario in this part of the salmon immune system. VRGs cover three tiers of immune responses: pathogen detection, signal transduction, and effector containing 3%, 15%, and 82% genes, respectively (**Figure 2B**). Although various pathogen recognition receptors have been identified in Atlantic salmon (28), only RNA helicases *rig1* (2 genes), *lgp2*, and *dhx58* have consistently demonstrated strong transcription responses to viruses. Signal transducers include *irf1, irf3, irf7* (2 genes), *stat1* (4 genes), and the lesser-known *srk2*. It should be noted that fewer terms are enriched in VRG than in BACT and INFL (**Table 2**), indicating that most VRGs are highly specialized for antiviral defense. Enrichment analysis indicated the key effector mechanisms associated with ubiquitin metabolism, histone binding, and lipid particles. Further inspection outlined other functional groups: metabolism of nucleotides and RNA and GTPase-mediated activity (**Figure 2C**), the functions of at least twenty salmon VRG are unknown.

**Figure 3:**
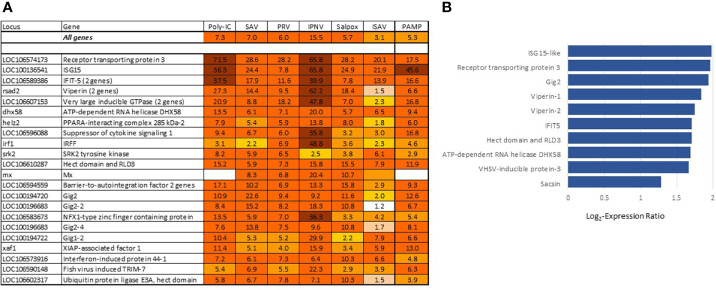
**(A)** Genes with the strongest antiviral responses. Here and in subsequent heat maps, data are the mean folds to intact controls. Information on tissues, cells and time-points is in **Table 1**. For each virus and poly(I:C) all data (contrasts) were averaged. **(B)** highly ranked VRG with preferential expression in erythrocytes (microarray data), log_2_-Expression Ratios to means of nine tissues and cell types.

The most differentially expressed VRG are in **Figure 3A**. About half of these genes have been investigated and characterized, but the roles of VRG that respond to viruses only in fish are much less known. This is true for *receptor transporting protein 3 (rtp3)*, VRG with the highest rank in Atlantic salmon showing strong responses to viruses in different fish species, including phylogenetically distant sturgeons (34, 35); its mammalian ortholog is involved in olfaction. Gene ranking improves the credibility of diagnostics: up-regulation of highly specialized VRG from the core set strongly suggests a viral infection. Such samples are clearly visible in transcriptome analyses. VRG are active in all tissues, although several highly ranked genes show elevated expression in erythrocytes (**Figure 3B**) in line with the important role of these cells in antiviral defense (6, 36). The increase in their transcripts may happen due to the stimulation of blood circulation, but the scale is much lower in comparison with infections. In theory, diagnostics can also be confounded with responses to bacterial DNA and TLR agonists. However, stimulation was observed in experiments with exposure of cells to PAMP (9) and muscle injection of plasmid DNA (12, 13) at doses that are probably never reached in naturally infected salmon. Several VRG were upregulated in our studies of pathogen-free inflammation but overlap with BACT was minor. RNA and DNA (salpox) viruses and dsRNA induce expression changes that are similar by magnitude (**Figure 3A**). The bias for the two pathogens with smaller (ISAV) and larger (IPNV) changes may be due to small datasets.

### Bacterial Diseases and Inflammation

**Figure 4:**
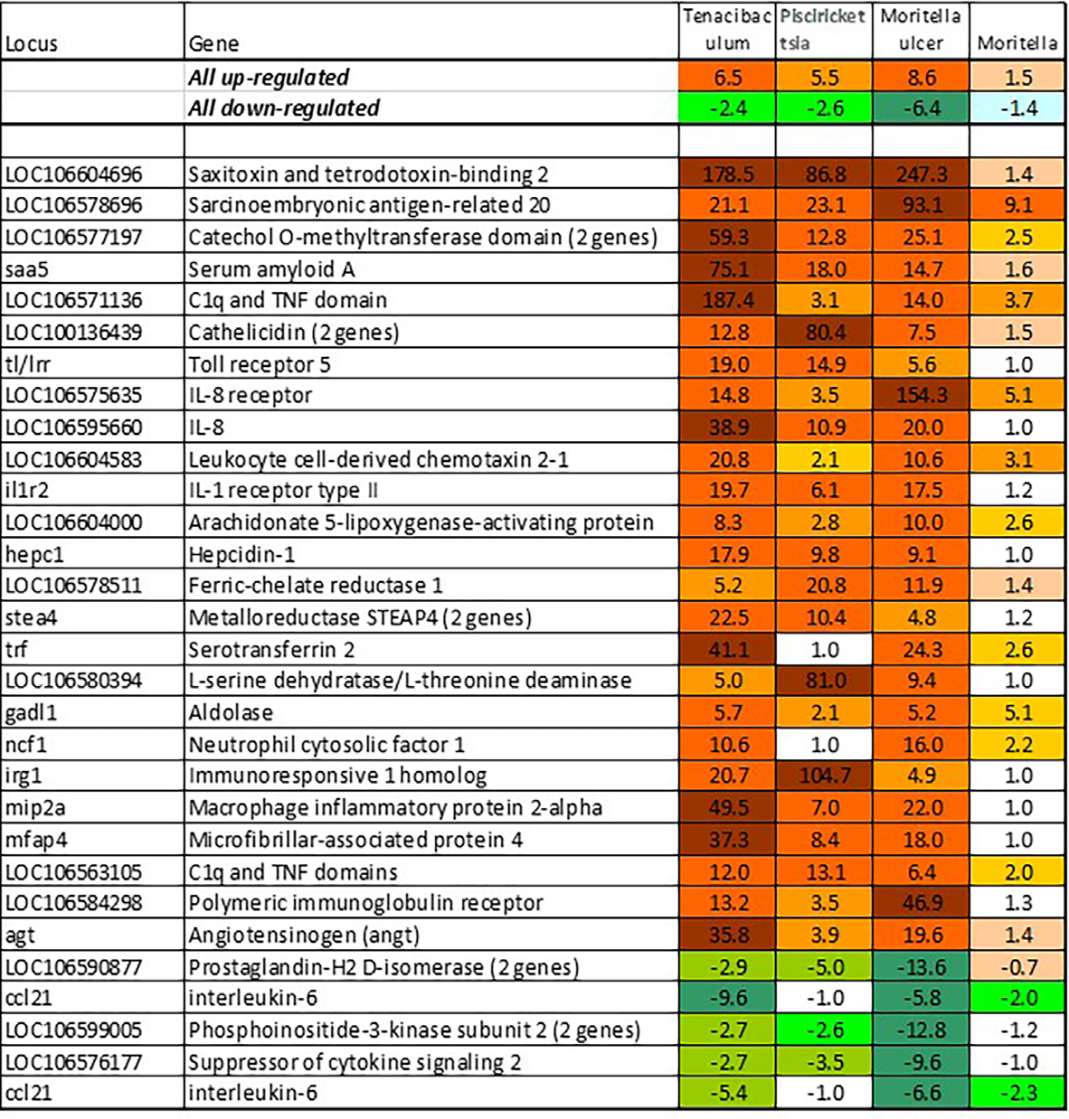
Responses to pathogenic bacteria: genes ranked by the expression changes with respect to uninfected control. Information on tissues, cells and time-points is in **Table 1**. Responses to *T. finnmarkense* and *M. viscosa* in whole skin and skin layers were averaged, ulcer is presented separately.

**Figure 5:**
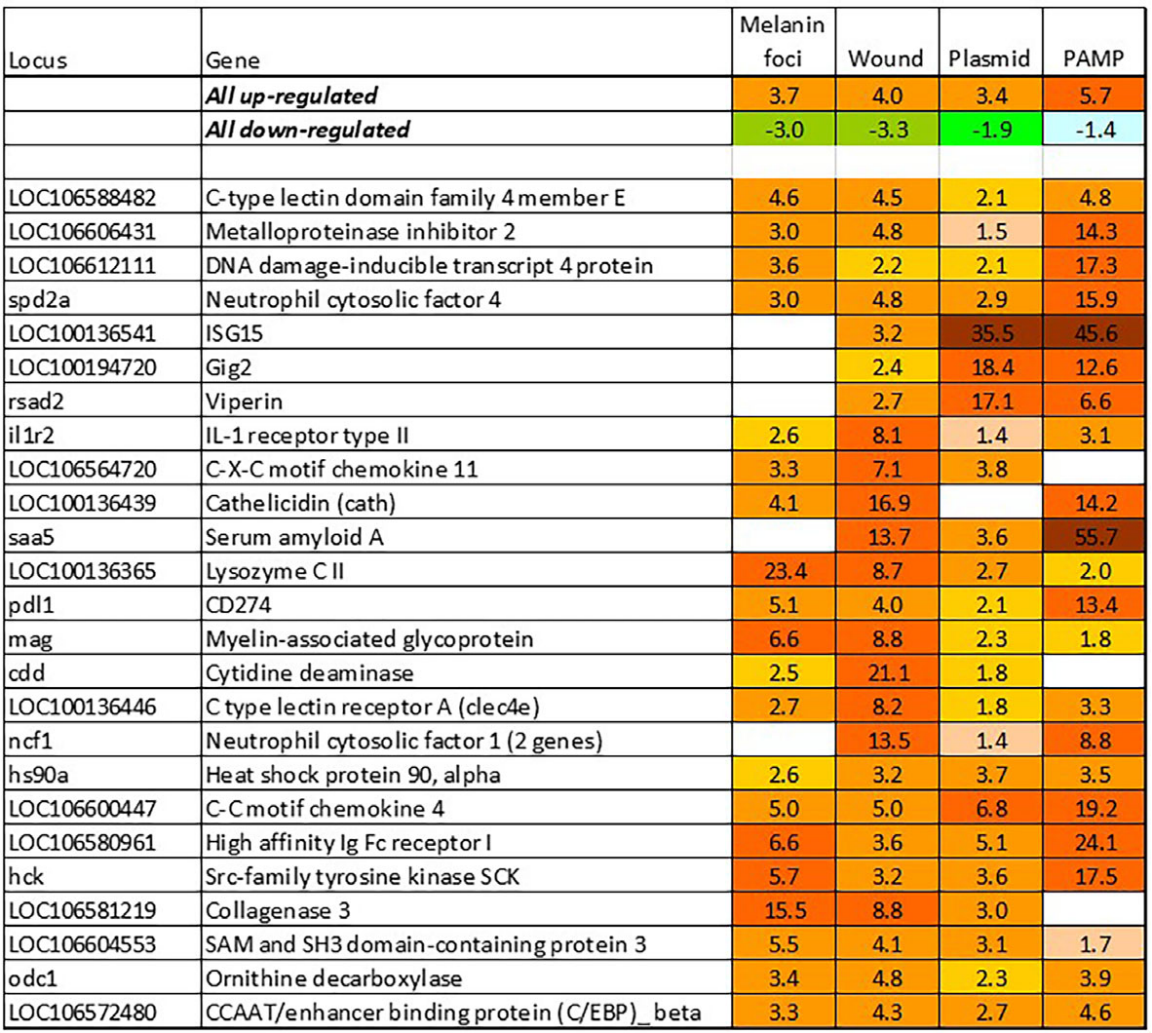
Responses to inflammation not caused by pathogenic bacteria. Genes are ranked by the expression changes. Information on tissues, cells and time-points is in **Table 1**.

BACT includes genes involved in stable and reproducible responses to bacterial pathogens that are manifested at high infection loads. Expression changes comparable with those induced with *T. finnmarkense* and *P. salmonis* were observed in skin ulcers caused by *M. viscosa*, but not in less severely damaged tissues (**Figure 4**). Responses are similar by scale, or even stronger, than those caused by viruses. Unlike VRG, a suite of genes with the highest ranks either have unknown roles (*saxitoxin and tetrodotoxin-binding protein* and *catechol o-methyltransferase domain* – number one and three in the list) or have not been reported in association with salmon diseases, for example, cell adhesion molecule *ceacam20* (37) and regulator of immune metabolism *irg1* (*aconitate decarboxylase)* (38). Both enrichment analysis and the gene composition of BACT indicate high complexity and diversity of responses to bacteria. TM includes a homolog of bacteria flagellin activated *tlr5* (39), chemokines, cytokines and receptors, and genes involved in the metabolism of lipid mediators, extracellular proteins, such as emblematic markers of inflammation *serum amyloid a* and *cathelicidin* and less investigated *c1q* and *tnf domain* proteins, members of a large multi-gene family, which has been known mainly in association with adipocyte differentiation and obesity (40). *Mmps* and enzymes of the polyamine/nitric oxide metabolism are presented as universal markers in **Figure 1B**. The importance of iron sequestration as an antibacterial defense strategy (41) is supported with strong responses of *hepcidin*, a master regulator of iron metabolism also known for antibacterial activity (42), extracellular iron transporter *transferrin*, and *steap4* – a TNFa-induced regulator of iron and copper homeostasis (43). In addition to immune genes, highly ranked BACT genes include enzymes of amino acid (*l-serine hydratase*) and sugar (*aldolase*) metabolism and regulator of blood pressure *angiotensinogen*. **Figure 4** presents immune genes with decreased expression. Down-regulation of *il-6* and *socs2*, genes with anti-inflammatory action, may enhance inflammation. The similarity of inflammatory responses caused by bacterial infections and non-pathogenic factors is suggested with enrichment of the same functional categories and pathways (**Table 2**). However, slightly more terms were significantly enriched in BACT, mainly due to the larger number of genes. Highly ranked INFL genes (**Figure 1B** and **Figure 5**) include chemokines, cytokines and their receptors, antibacterial and acute phase proteins (*lysozyme*, *cathelicidin* and *serum amyloid a*), *lectins* and *Ig receptor*, ROS producing *neutrophil cytosolic factors*, enzymes of polyamine and nitric oxide metabolism, *matrix metalloproteinases* and *mmp inhibitor*. In addition to *mmp 9* and *13*, strong expression changes were shown by the fish-specific *collagenase-3 like* enzyme. Wound up-regulated several VRG (*isg15*, *viperin* and *gig2*). The roles of highly ranked *cytidine deaminase* and *myelin-associated glycoprotein* in inflammation remain unknown. Overall, pathogen-free inflammatory responses included fewer genes than those caused by disease-causing bacteria, but the changes in expression were comparable in scale.

### Stress Response

**Figure 6:**
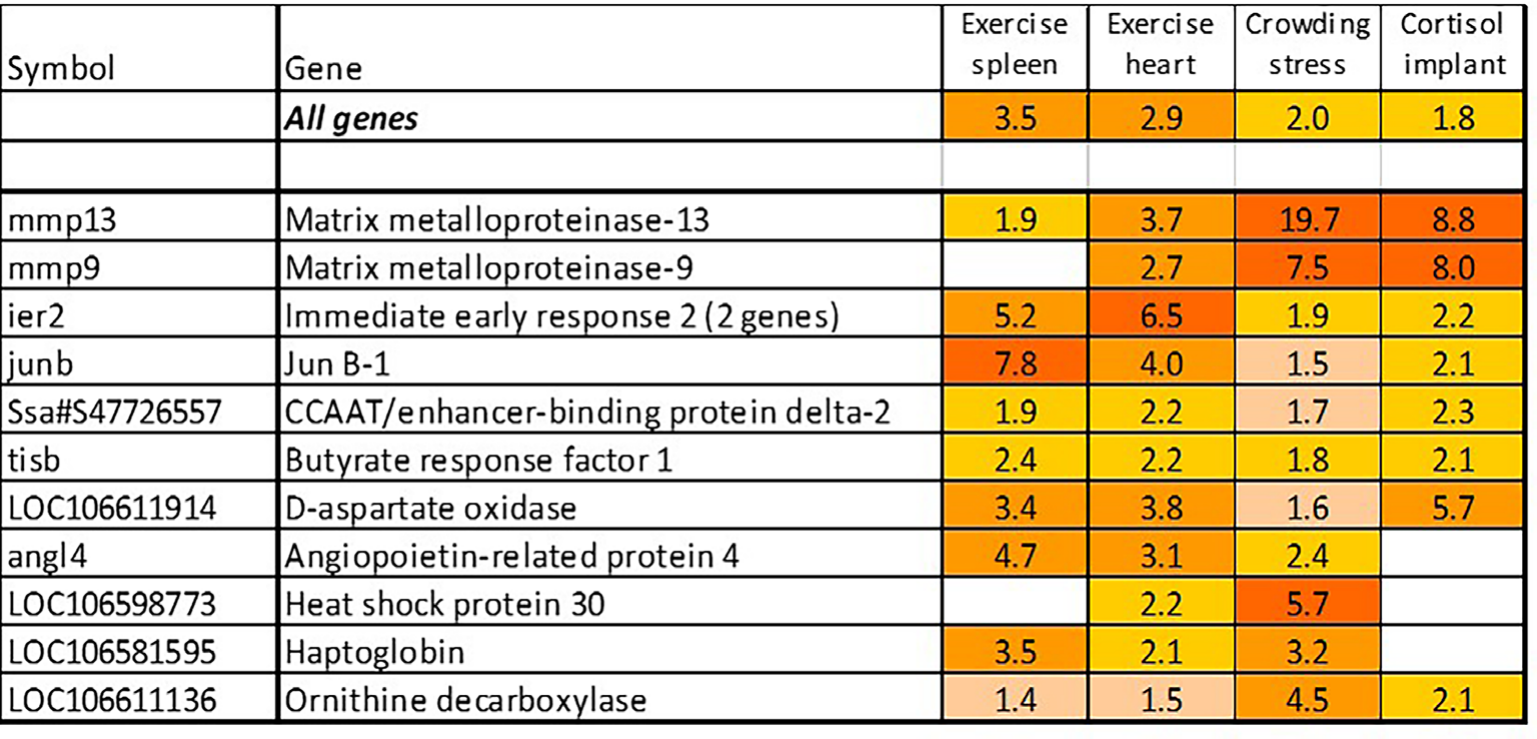
Stress genes. Information on tissues, cells and time-points is in **Table 1**.

The term “stress” has different meanings depending on the context, and it is hardly possible to find universal stress markers. We have identified a panel of genes activated with pathogens and inflammatory agents, which were also induced with treatments not related to any infectious disease and pathology, such as acute exercise and crowding, and most of these genes also responded to cortisol, the main endocrine mediator of stress in salmonid fish (**Figure 6**). Inspection of these trials found several more consistently up-regulated genes that were added to stress TMs. These TMs make quantitative evaluation of stress components under different conditions possible. Of genes not shown in **Figure 1B**, association with stress was known only for *hsp30*, a chaperone not found in mammals (44), and DNA binding *immediate early response – ier2* [cold-induced in embryos of the kelp grouper, *Epinephelus moara* (45)]. *Butyrate response factor – brf1*, another early response gene, regulates the decay of mRNA (46). Free heme neutralizing *haptoglobins* respond to chemical stress in Atlantic salmon (47). *Angiopoietin-related protein 4* stimulates angiogenesis under hypoxic conditions (48) and the role of *d-aspartate oxidase* in stress responses is unknown.

### Separation of the Transcriptome Into Fractions and the Contribution of TMs

**Figure 7:**
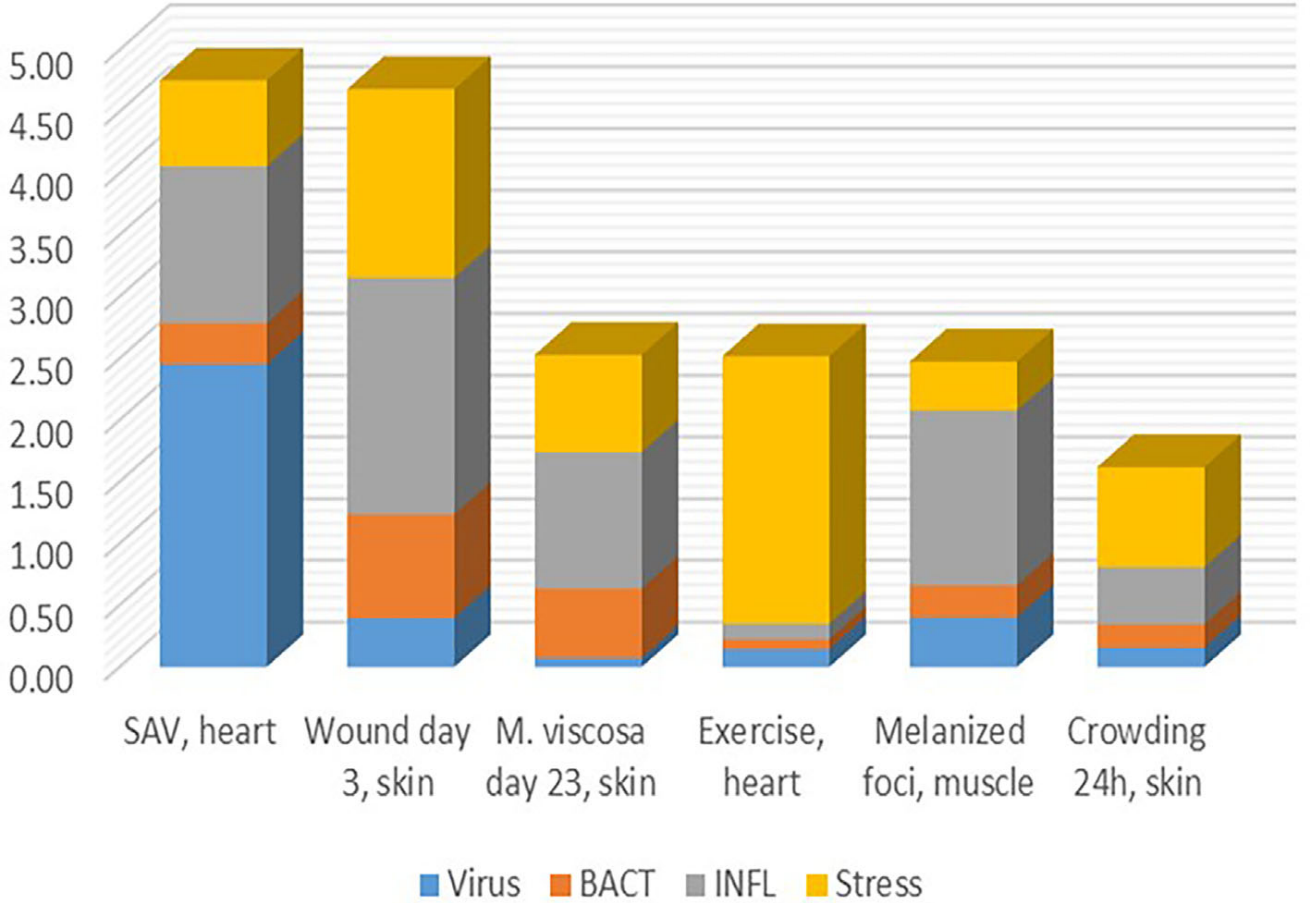
Examples of transcriptome responses (contrast) with contribution of several TM shown as the mean log_2_-Expression Ratios.

Finding thematic associations with the help of functional annotations is probably the most commonly used approach to the analysis and biological interpretation of complex transcriptome responses. Its weakness lies in the implicit assumption of similar expression profiles of functionally related genes. However, this information is not available in public databases. Genes with similar molecular roles can be activated under different conditions (then co-expression is not observed) and many genes generally do not show responses at the transcription level. The identification of TM significantly enhances this approach by providing groups of genes, the responses and co-regulation of which have been confirmed on a large experimental basis. Pure cases are rare, and as a rule, transcriptome responses include several components with different contributions (**Figure 7**). This may help to understand poorly explored problems, such as melanized foci (dark spots) in the skeletal muscle causing heavy economical losses in Atlantic salmon aquaculture, the origin of which has been debated (14, 49). Judging by the minor contribution of antiviral and antibacterial responses, one may think a non-infectious factor probably causing them. STR allows to assess the degree of stress caused with various disturbances including infection with pathogens, wounds, and other damages. Given that a linear gene expression response to temperature has been shown (50, 51), graded effects of other stressors can be expected.

## Discussion

The potential of transcriptome data for the functional annotation of genes and discovery of biomarkers is obvious, and rapid progress in this area can be expected. The reported approach is based on the search of groups of co-regulated Atlantic salmon genes referred to as transcription modules – TMs. Our meta-analyses have revealed two types of TM. The first type includes gene sets showing consistently high correlation of expression profiles most likely due to transcriptional co-regulation, examples are Atlantic salmon myofiber proteins co-regulated with proteins of calcium and glucose metabolism, pituitary hormones, immunoglobulins, and genes involved in steroid biosynthesis (not included in this paper). The number of such groups is small and each gene is firmly anchored to only one TM. Here, we present genes that have shown differential expression under certain conditions, where each member of the TM can be relatively independent of the rest and belong to several groups. We focused on responses to bacterial and viral pathogens, inflammatory agents, and stress, due to the high importance of these issues for aquaculture and the large amount of data produced by our group. A similar search can be implemented for any biological process and trait, and the only requirement is a set of high-quality transcriptome data. In our approach, the main priority is the consistency of expression changes – each gene in the TM must show differential expression in several independent trials under the standard thresholds. The results of transcriptome analysis in similar studies, as a rule, do not coincide in detail, and even if the main trends are stable, the deviations can be large. This does not mean a weakness in the design of experiments and analysis. The instability is largely determined with the complexity and diversity of transcriptome responses combined with an imbalance between the number of measurements (genes) and the number of animals, known as the multiplicity problem. There can be several different scenarios for responding to a challenge in a population. A small sampling group can highlight genes or groups of genes that are affected in some individuals but will not overcome statistical thresholds of significance in a representative comparison of populations. Deviations between experiments can also be caused by the difference in the genetic background, age and physiological condition of fish, season, the severity of infections and damages and many other factors. Both annotations and diagnostics are based on stable responses and therefore, a meta-analysis of independent studies is essential.

The four presented TMs include many uncharacterized genes and genes whose association with responses to pathogens, inflammation and stress was unknown or not reflected in public databases. In addition, differential expression and its scale can add a new dimension to the functional annotations. The composition of TMs is useful for the understanding of protective strategy in Atlantic salmon. VRG represents a separate branch of innate immunity characterized with relatively high specificity, although they can be activated with abnormally high levels of PAMP. The similarity of responses to bacteria and non-pathogenic inflammation suggests the dominance of proactive defense in Atlantic salmon. The mission of inflammation is the clearance of infection and damaged tissues – cell debris and extracellular matrix. We see that even in the absence of infections, inflammation includes a powerful effector arm targeted at killing microorganisms. In our studies, experimentally inflicted wounds induced inflammatory responses, which were similar to bacteria-caused ulcers being much stronger in comparison with the skin of salmon infected with *M. viscosa.* The stress component is clearly seen in all treatments including the viral and bacterial infections and inflammation caused by non-pathogenic factors. Identification of TM enhances diagnostics. In relation to transcriptomic data, differential expression of genes representing a significant portion of TM substantially increases the confidence of conclusions and ranking of genes provides additional validation: upregulation of the core set is strong evidence. TMs allow quantification and comparison of the magnitude of responses. TMs also serve as a basis for developing biomarkers and diagnostic sets of genes for qPCR or multigene assays, such as our assay of immune competence (3, 8, 8, 52) developed on the Biomark HD platform, which was recently introduced in research with salmonid fish (31, 50, 53). These studies validated differential expression under various conditions and determined the core gene sets for various diagnostic tasks.

TMs are open to changes and improvements and new groups of co-expressed genes will be identified. The core set of VRG has been well established: we have worked with all important viral infections of concern to the Atlantic salmon industry and did not find significant differences in responses. Although no major changes are expected even if new pathogens will emerge, low-ranked VRG can be added or excluded with new experimental data. Some genes can either exceed or fall below the threshold value, but the adjustments are unlikely to go beyond fine-tuning. INFL is mainly based on studies of wound healing with support from melanized foci and exposures to PAMP. New trials with strong inflammation, free of pathogens, would be very useful to verify and strengthen this association. We can expect certain changes in the set of genes activated with pathogenic bacteria. We currently have extensive data sets for *T. finnmarkense* and *M. viscosa* only. High variation has been observed with respect to the disease stage and the severity of lesions, especially for *M. viscosa*. In the future, it may be necessary to split BACT or compile several groups of genes activated or suppressed with bacterial infections. The same is true for STR, because this TM was created to highlight the stress component in the challenge trials and responses that otherwise had little in common. Most genes included in TMs are highly expressed in different organs or in blood cells that infiltrate infected or damaged tissues. Currently, we do not have enough material to search for genes with tissue-specific responses. Related task is discovery of markers discriminating responses to specific stressors and pathogens (31, 50) and differential diagnostics of diseases (54, 55). Here, we report the first step in using transcriptome data for functional annotation of Atlantic salmon genes in order to demonstrate the usefulness and potential of this approach.

## Data Availability Statement

Publicly available datasets were analyzed in this study. This data can be found here: https://www.ncbi.nlm.nih.gov/geo/subs/GSE172862 GSE171562 GSE171693 GSE171738 GSE173130 GSE171699 GSE173095 GSE173119 GSE173229 GSE183005 GSE183260 GSE183265.

## Author Contributions

AK – conceptualization, methodology, original draft preparation. SA – software, data curation. L-HJ, CK, LS, EY, GT, CL – experiments and laboratory analyses, writing. All authors contributed to the article and approved the submitted version.

## Funding

The study was funded by the National Research Council of Norway (267644) and Nofima (internal grant). SA was supported with a grant from I. M. Sechenov Institute of Evolutionary Physiology and Biochemistry (IEPHB RAS, research theme No. АААА-А18-118012290373-7).

## Conflict of Interest

The authors declare that the research was conducted in the absence of any commercial or financial relationships that could be construed as a potential conflict of interest.

## Publisher’s Note

All claims expressed in this article are solely those of the authors and do not necessarily represent those of their affiliated organizations, or those of the publisher, the editors and the reviewers. Any product that may be evaluated in this article, or claim that may be made by its manufacturer, is not guaranteed or endorsed by the publisher.

## Supplementary Material

Supplementary File Contains two sheets. List – all annotated genes with ranks in TMs. Data – contrasts used for meta-analyses. Microarrays contain different probes to the same genes; identifiers are in columns C and D. Data are contrasts (log_2_-expression ratios of treated to controls). Rows 3 – identifiers, line 4 – source (cells, tissues and origin), line 5 – treatment. Missing and insignificant data (no differential expression) are highlighted with colour.
